# Thermodynamics-like Formalism for Immiscible and Incompressible Two-Phase Flow in Porous Media

**DOI:** 10.3390/e27020121

**Published:** 2025-01-24

**Authors:** Alex Hansen, Santanu Sinha

**Affiliations:** PoreLab, Department of Physics, Norwegian University of Science and Technology NTNU, N-7491 Trondheim, Norway; santanu.sinha@ntnu.no

**Keywords:** flow in porous media, immiscible two-phase flow, co-moving velocity

## Abstract

It is possible to formulate an immiscible and incompressible two-phase flow in porous media in a mathematical framework resembling thermodynamics based on the Jaynes generalization of statistical mechanics. We review this approach and discuss the meaning of the emergent variables that appear, agiture, flow derivative, and flow pressure, which are conjugate to the configurational entropy, the saturation, and the porosity, respectively. We conjecture that the agiture, the temperature-like variable, is directly related to the pressure gradient. This has as a consequence that the configurational entropy, a measure of how the fluids are distributed within the porous media and the accompanying velocity field, and the differential mobility of the fluids are related. We also develop elements of another version of the thermodynamics-like formalism where fractional flow rather than saturation is the control variable, since this is typically the natural control variable in experiments.

## 1. Introduction

In spite of the immiscible multi-phase flow in porous media being a field of great industrial importance for more than a century, basic research in this field has not received the attention that it deserves. The main reason for this is that the field is fragmented, being spread over many different disciplines such as biology, geology, geophysics, physics, chemistry, and materials science. Another reason is that it is easy to get the impression that it is not “clean” in the sense that it is a field where theories may be built. Rather, it seems to be a collection of phenomena in need of classification. It is the aim of this paper to convince the readership that this is indeed not the case. There is a physics of flow in porous media [1] where general principles may be formulated. We will, in the following, present the readers with a review of our ongoing attempts at formulating a mathematical framework for immiscible and incompressible two-phase flow in porous media.

In 1907, Buckingham introduced several of the central concepts used today in describing immiscible and incompressible fluid flows in porous media [2,3], but without joining them through equations. This was first performed by Richards in 1931 [4] who, as Buckingham, placed the flow problem in the context of water movement in soils. In 1936, building on the work by Wyckoff and Botset [5], Muscat and Meres [6] introduced the concept of relative permeability, a concept generalizing the notion of “capillary conductance” that Buckingham introduced [2]. With the introduction of the capillary pressure curve by Leverett [7] into the framework established by Muscat and Meres, the generalized Darcy constitutive equations that are generally used today in, e.g., reservoir simulation, were in place. The formulation of the problem in the context of water in soils that Buckingham pioneered, from the work of Richards, forms an alternate formulation of essentially the same ideas.

We review briefly relative permeability theory—the only approach to immiscible two-phase flow in porous media in practical use today—in Section 2. Relative permeability theory is purely phenomenological, and has well-known deficiencies. As a result, there is an ongoing effort to derive effective equations going beyond relative permeability theory based on the physics at the pore scale. This effort is based on *homogenization* which we review briefly in Section 3.

In Section 4, we introduce our approach to immiscible two-phase flow in porous media based on a generalization of statistical mechanics by Jaynes [8,9]. Statistical mechanics in its traditional role is to connect a molecular description of matter to a description at the much larger scales where matter appears continuous, namely thermodynamics. In adopting this idea to two-phase flow in porous media, we find a thermodynamics-like framework on large scales that together with conservation laws provide a closed set of equations that has the equations of relative permeability theory as a special case. As in ordinary thermodynamics, the difference between intensive and extensive variables is central. We explore this in Section 5. The intensive variables appearing in the thermodynamics-like formalism we derive are emergent in that they have no equivalent on the pore scale. This is discussed in Section 6. The temperature-like intensive variable, *agiture*, we conjecture to be proportional to the pressure gradient in Section 6.1. This conjecture has as a consequence in that the *configurational entropy*, which is an example of Shannon’s information entropy, is related to the differential mobility of the fluids. The differential mobility is the derivative of the flow velocity with respect to the pressure gradient, and the mobility is the flow velocity divided by the pressure gradient. The *flow derivative* we discuss in Section 6.2 is the conjugate variable to the saturation, which is the fractional volume. We end the section by discussing the conjugate of the porosity, the *flow pressure*, in Section 6.3.

In Section 7, we introduce the *co-moving velocity* which relates the thermodynamic velocities—corresponding to partial molar volumes in ordinary thermodynamics—to the seepage velocities, which are the average pore velocity of each of the immiscible fluids. This concept has the potential to become a very useful concept in practical calculations.

Section 8 contains material that has not been presented before. We start by pointing out a choice made when constructing the Jaynes statistical mechanics in Section 4, and how it could have been made differently. Then we discuss what the ensuing thermodynamics-like formalism would be as a consequence: rather than having saturation as one of the control parameters, we find fractional flow rate being a control parameter. This is useful, as this is the control parameter used in core flooding experiments.

We end by summarizing and concluding in Section 9.

## 2. Relative Permeability Theory

The core idea of the relative permeability approach may be summarized as follows: We have two immiscible fluids, one more wetting with respect to the porous matrix than the other fluid. We will refer to them as the wetting (w) and non-wetting (n) fluid, respectively. From the perspective of the wetting fluid, the pore space it sees is the total pore space of the porous medium minus the pore space occupied by the non-wetting fluid, and vice versa. The relative reduction of pore space for each fluid implies a reduction in effective permeability for each fluid. We express these statements in the form of two constitutive equations, one for each fluid [1],

(1) $$\begin{array}{ccc} {\vec{v}}_{w} & = & -\;\frac{Kk_{rw}\left(S_{w}\right)}{\mu_{w}\phi S_{w}}\nabla P_{w}\;, \end{array}$$

(2) $$\begin{array}{ccc} {\vec{v}}_{n} & = & -\;\frac{Kk_{rn}\left(S_{w}\right)}{\mu_{n}\phi S_{n}}\nabla P_{n}\;. \end{array}$$

Here, ${\vec{v}}_{w}$ and ${\vec{v}}_{n}$ are the pore velocities of the wetting and non-wetting fluids, *K* is the (isotropic) permeability of the porous medium, $\mu_{w}$ and $\mu_{n}$ are the viscosities of each fluid, ϕ is the porosity of the porous medium, $P_{w}$ and $P_{n}$ are the fluid pressures, $S_{w}$ and $S_{n}$ are the wetting and non-wetting saturations, and $k_{rw}\left(S_{w}\right)$ and $k_{rn}\left(S_{w}\right)$ are the relative permeabilities of the two fluids. It is an essential *assumption* in this theory that these two quantities are functions of the saturations alone. The saturations $S_{w}$ and $S_{n}$ are defined as the fraction of pore space occupied by each fluid so that

(3) $$S_{w}+S_{n}=1\;.$$

The difference in pressure between the two fluids is defined as the capillary pressure curve $P_{c}$,

(4) $$P_{n}-P_{w}=P_{c}\left(S_{w}\right)\;.$$

It is an assumption that the capillary pressure curve only depends on the saturation $S_{w}$. The capillary pressure curve is a particularly difficult quantity, both conceptually and in terms of measurement (see, e.g., [10]). We define the average pore velocity ${\vec{v}}_{p}$ as

(5) $${\vec{v}}_{p}=S_{w}{\vec{v}}_{w}+S_{n}{\vec{v}}_{n}\;,$$

i.e., we are using a volume average. This makes sense since we are assuming the fluids to be incompressible,

(6) $$\nabla \cdot [\phi {\vec{v}}_{p}]=0\;.$$

Volume conservation then gives

(7) $$\begin{array}{ccc} \phi \frac{\partial S_{w}}{\partial t}+\nabla \cdot \left[\phi S_{w}{\vec{v}}_{w}\right] & = & 0\;, \end{array}$$

(8) $$\begin{array}{ccc} \phi \frac{\partial S_{n}}{\partial t}+\nabla \cdot \left[\phi S_{n}{\vec{v}}_{n}\right] & = & 0\;, \end{array}$$

where *t* is time. This set of equations is closed as long as $k_{rw}\left(S_{w}\right)$, $k_{rn}\left(S_{w}\right)$, and $P_{c}\left(S_{w}\right)$ are provided. In an industrial context, Special Core Analysis (SCAL) forms the work flow for obtaining this information.

## 3. Homogenization

It is well known that assuming the three constitutive functions depend only on the saturation is generally not correct. The relative permeabilities do depend not only on the saturation, but also how the fluids arrange themselves in the porous medium, and this depends, in turn, on the flow rate. The capillary pressure curve is highly hysteretic, signaling that there are missing variables. However, it has turned out to be difficult to go beyond this almost-90-year-old phenomenological framework. The dominating approach is based on homogenization. This approach may be divided into two branches: that which focuses on momentum transfer, and that which focuses on energy transfer. In the first case, one starts with the hydrodynamic equations on the pore scale [11,12,13,14,15,16], and in the second case, one sets up a thermodynamic description at the pore scale [17,18,19,20]. Then comes the coarse-graining, i.e., spatial averaging. There are several ways of performing this; perhaps that of Whitaker is the most well-known [21,22]. It is based on equating the average of the gradient of a variable associated with pore space to the gradient of the average variable plus an integral over the surface area of the pores. Since the surface area of porous media scales as the volume (i.e., the surface area is *extensive*, the defining property of porous media), this integral does not vanish as one goes up in scale. We split the variable appearing in the surface integral into an average part and a fluctuating part. This leaves us with having the average, and gradients of the average expressed in terms of the fluctuations of the original variable. The last step is to make an independent assumption on how the fluctuating variables is related to the averages—a closure assumption. With this, the equations between the original, pore-scale variables have been turned into equations between spatial averages of these variables.

Whereas the first of these two approaches is based on momentum transfer, the second approach is based on energy transfer, i.e., thermodynamics. One starts with a thermodynamic description on the pore level which is then homogenized. This approach has developed into Thermodynamically Constrained Averaging Theory (TCAT) [23,24,25,26].

A recent paper by McClure et al. [27] attempts to derive the relative permeability equations from an energy budget based on thermodynamic considerations and homogenization. The relative permeability equations do appear as a first term in a series expansion. However, it is not shown in [27] that the higher order terms are negligible.

The topology of a geometric object such as a porous medium may be described using the four *Minkowski functionals:* volume, surface area, mean curvature, and the Euler characteristics. The *Hadwiger theorem* states that the Minkowski functionals form a complete basis set for all extensive functions that are invariant with respect to the orientation of the object [28]. The use of this theorem to characterize the free energy of fluids in a porous medium combined with homogenization forms another approach to the scale-up problem [29,30,31,32]. Homogenization as seen so far is based on *spatial* averages only. McClure et al. [33,34] emphasize that there is also time which one should average over and that different processes work on different time scales.

An approach circumventing the complexities associated with homogenization is based on classical non-equilibrium thermodynamics [35,36,37,38,39,40]. By using the extensiveness of the internal energy of the fluids in the porous medium, the Euler theorem for homogeneous functions allows for defining thermodynamic variables such as pressure and chemical potentials on the Darcy scale. Gradients in the intensive variables are introduced, and the machinery of classical non-equilibrium thermodynamics [35,36] is then set in motion. The underlying homogenization is somewhat hidden in this approach, but it is underlying the way a Representative Elementary Volume (REV) is defined and used.

The main practical difficulty with homogenization is the complexity of the ensuing equations and the large number of variables that are necessary. The root of this difficulty is that homogenization can only produce averages over the original variables, and thus cannot produce new types of variables which go hand-in-hand with capturing emergent properties [41].

## 4. Statistical Mechanics

The essence of these efforts has been to find a description of immiscible and incompressible two-phase flow in porous media at scales where the medium acts as a continuum. Including compressibility would necessitate switching from conserved fluid volume to conserved fluid mass. It would also couple the theory to ordinary thermodynamics through equations of state for the compressible fluids. We wish to avoid these complications at the present stage. This is an example of *upscaling*. The situation is thus that we have a continuum scale phenomenological theory; a relative permeability theory that is simple enough to be used in practical situations, but which is approximate. Attempts at going beyond this theory has, however, not led to practical applications due to the complexity that arises. However, if we look beyond the problem we discuss here, there is an example of successful upscaling that is very familiar: The scale-up from a molecular description to thermodynamics based on statistical mechanics. At molecular scales, the position and momentum of each molecule are the variables that describe a monoatomic gas. This works even if we are dealing with millions of molecules in a molecular dynamics simulation [42]. However, it is rather of the order $10^{23}$ molecules that are relevant in, e.g., chemistry. A molecular description at such scales is useless. Here, *emergent variables* such as temperature and pressure adequately describe the gas. Statistical mechanics converts the molecular description to thermodynamics which not only contains emergent variables, but also relations between them. These variables and their relations have no meaning within a molecular description.

In the mid-fifties, Jaynes generalized statistical mechanics from being a theory for molecular matter to being based on information theory [8]. This was performed by replacing Boltzmann’s statistical interpretation of thermodynamic entropy by Shannon’s generalized information entropy [43]. The information entropy is a quantitative measure of what is *not* known about a system. Suppose we have a stochastic process that can produce *N* possible outcomes. We number the outcomes from 1 to *N*, assuming a probability $p_{i}$ for the *i*th outcome, $x_{i}$. A quantitative measure of our ignorance about this process must be independent of how we group events together. For example, if we consider composite events such as $p_{ij}=p_{i}p_{j}$, i.e., the probability that an event *i* is followed by an event *j*, then the information entropy must remain the same. If we know nothing about the process, its information entropy must be at a maximum, since our ignorance is maximal. In order to utilize this, Shannon generalized to *N* outcomes the Laplace principle of insufficient reason [44], which states that the optimal choice of probabilities for a stochastic process with *two* possible outcomes is to assign them equal probability. Hence, the optimal choice when there are *N* outcomes is to assign all probabilities the same value, $p_{i}=1/N$. There is only one function that can be constructed from $p_{i}$ with these properties,

(9) $$S_{I}=-\sum_{i=1}^{N}p_{i}\ln p_{i}\;,$$

which is then the information or Shannon entropy. Jaynes then posed the question: what happens if we *do* know something about the system? Suppose we know the average of the outcomes,

(10) $$\langle x\rangle =\frac{1}{N}\sum_{i=1}^{N}p_{i}x_{i}\;.$$

Generalizing the principle of insufficient reason further, Jaynes proposed that the probabilities $p_{i}$ in this case should be those that maximize the information entropy (9) given a fixed value for 〈x〉 (Equation (10)).

Jaynes provided a set of circumstances for his generalized statistical mechanics to be applicable. Hansen et al. [9] demonstrated that these circumstances are met for steady-state immiscible two-phase flow in porous media, and hence proceeded to construct such a statistical mechanics. The generalized statistical mechanics will produce a thermodynamics-like framework at large scales. This thermodynamic-like framework is the end goal of this approach.

**Figure 1:**
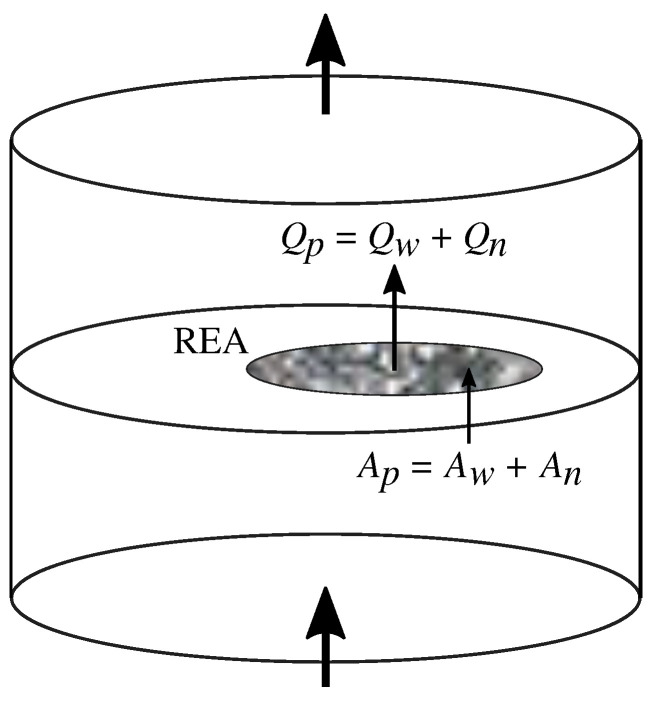
We define a plane that cuts through the cylindrical pore sample orthogonally to the average flow direction. We define Representative Elementary Area (REA) within the plane. There is a wetting fluid flow rate $Q_{w}$ and a non-wetting fluid flow rate $Q_{n}$ passing through the REA. The total flow rate is $Q_{p}$. The wetting fluid covers an area $A_{w}$ of the REA and the non-wetting fluid an area $A_{n}$. The total pore area of the REA is $A_{p}$.

Suppose we have a cylindrical pore sample as shown in Figure 1. Two immiscible fluids enter at the bottom and leave at the top. The side walls are close off. We assume that the pore structure and chemical composition of the matrix to be statistically uniform throughout the sample. At some distance from the lower edge of the sample, one may assume that the fluids have mixed sufficiently to assume steady-state flow. That is, if we make transversal cuts through the sample, the structure of the porous medium and the distribution of fluids in the pore space to be statistically uniform: Comparing one cut to another, we will not be able to determine which is closest to the inlet. Figure 1 shows one such cut. Within this cut, we pick out a *Representative Elementary Area* (REA), which is large enough to represent the statistics of the sample and small enough to have the flow fluctuate. There is an volumetric flow rate $Q_{p}$ passing through the REA which may be split into a volumetric flow rate of the wetting fluid $Q_{w}$ and the volumetric flow rate of the non-wetting fluid $Q_{n}$, i.e.,

(11) $$Q_{p}=Q_{w}+Q_{n}\;.$$

The area of the REA is *A*. An area $A_{p}<A$ cuts through pore space. This area may be divided into the area cutting through the pore space filled with wetting fluid, $A_{w}$ and the area cutting though the pore space filled with the non-wetting fluid, $A_{n}$, and we have that

(12) $$A_{p}=A_{w}+A_{n}\;.$$

At a given time for a given REA, the fluid configuration, characterized by the velocity field and where the fluids are, is *X*. The quantities that describe the REA depend on *X*, so that we have $Q_{p}\left(X\right)$, $Q_{w}\left(X\right)$, $Q_{n}\left(X\right)=Q_{p}\left(X\right)-Q_{w}\left(X\right)$, $A_{p}\left(X\right)$, $A_{w}\left(X\right)$, and $A_{n}\left(X\right)=A_{p}\left(X\right)-A_{w}\left(X\right)$. Of course, $A_{p}\left(X\right)$ is not dependent on the velocity field, nor the distribution of the two fluids, but only on the shape of pore space. Let us denote p(X) the probability density to find fluid and pore space configuration *X*. We define a *configurational entropy*,

(13) S=−∫dXp(X)lnp(X).

The probability density p(X) is *continuous*, so that we are dealing with differential entropy [45] in Equation (13). Another way of dealing with the transition from discrete to continuous systems is that of Jaynes [46].

We assume that we know the averages (in time and position of the REA) of the variables just described, i.e.,

(14) $$\begin{array}{ccc} Q_{u} & = & \int dX\;p\left(X\right)\;Q_{u}\left(X\right)\;, \end{array}$$

(15) $$\begin{array}{ccc} A_{w} & = & \int dX\;p\left(X\right)\;A_{w}\left(X\right)\;, \end{array}$$

(16) $$\begin{array}{ccc} A_{p} & = & \int dX\;p\left(X\right)\;A_{p}\left(X\right)\;. \end{array}$$

The variable $Q_{u}$ is related to the volumetric flow rate $Q_{p}$, but we will defer its definition until Equation (30).

Following the Jaynes maximum entropy principle, we maximize the entropy (13) given the constraints (14) to (16), finding that

(17) $$p\left(X;\lambda_{u},\lambda_{w},\lambda_{p}\right)=\frac{1}{Z(\lambda_{u},\lambda_{w},\lambda_{p})}\;\exp\left[-\lambda_{u}Q_{u}\left(X\right)-\lambda_{w}A_{w}\left(X\right)-\lambda_{p}A_{p}\left(X\right)\right]\;,$$

where

(18) $$Z\left(\lambda_{u},\lambda_{w},\lambda_{p}\right)=\int \;dX\;\exp\left[-\lambda_{u}Q_{u}\left(X\right)-\lambda_{w}A_{w}\left(X\right)-\lambda_{p}A_{p}\left(X\right)\right]$$

is the normalization factor, also known as the partition function. Three new *emergent variables* have appeared, $\lambda_{u}$, $\lambda_{w}$, and $\lambda_{p}$. We write the partition function as

(19) $$Z\left(\lambda_{u},\lambda_{w},\lambda_{p}\right)=\exp\left[-\lambda_{u}Q_{z}\left(\lambda_{u},\lambda_{w},\lambda_{p}\right)\right]\;.$$

The partition function is in some sense the “end product” of a statistical mechanics approach to a problem. It is the generating function for the probability distribution for microscopic configurations. With it, all macroscopic averages may be calculated.

In ordinary statistical mechanics, a Hamiltonian, i.e., a microscopic energy function is necessary input for calculating the partition function. In the present problem we have three pore-level functions, $Q_{u}\left(X\right)$, $A_{w}\left(X\right)$ and $A_{p}\left(X\right)$. The problem in ordinary statistical mechanics, is that even for seemingly simple problems such as the two-dimensional Ising model for magnetic systems, calculating the partition function may be extremely difficult (cfr. Onsager’s calculation of the partition function for this system [47]). The problem is surely no less difficult in the porous media context. Perhaps, it is even worse in that we do not have good models for the three microscopic functions in contrast to ordinary statistical mechanics where model Hamiltonians are legio.

A byproduct of the statistical mechanics approach is the emergence of a set of thermodynamics-like relations between the REA-scale variables. This framework will be in place whether it is possible to calculate the partition function or not. We will now develop this thermodynamics-like framework for immiscible two-phase flow in porous media.

We start by calculating the entropy *S* by inserting Equation (17) in Equation (13) and using Equation (19). We obtain

(20) $$S\left(Q_{z},A_{w},A_{p}\right)=-\lambda_{u}Q_{z}\left(\lambda_{u},\lambda_{w},\lambda_{p}\right)+\lambda_{u}Q_{u}+\lambda_{w}A_{w}+\lambda_{p}A_{p}\;.$$

We rewrite this equation as

(21) $$Q_{z}=Q_{u}-\frac{1}{\lambda_{u}}\;S+\frac{\lambda_{w}}{\lambda_{u}}\;A_{w}+\frac{\lambda_{p}}{\lambda_{u}}\;A_{p}\;.$$

It is convenient to define the new variables

(22) $$\begin{array}{ccc} \theta & = & +\frac{1}{\lambda_{u}}\;, \end{array}$$

(23) $$\begin{array}{ccc} \mu & = & -\frac{\lambda_{w}}{\lambda_{u}}\;, \end{array}$$

(24) $$\begin{array}{ccc} \pi & = & -\frac{\lambda_{p}}{\lambda_{u}}\;. \end{array}$$

We may then write Equation (21) as

(25) $$Q_{u}\left(S,A_{w},A_{p}\right)=Q_{z}\left(\theta ,\mu ,\pi \right)+\theta S+\mu \;A_{w}+\pi \;A_{p}\;,$$

where we have used that this is a Legendre transform (assuming convexity of the involved functions), since

(26) $$\begin{array}{ccc} S & = & -{\left(\frac{\partial Q_{z}}{\partial \theta }\right)}_{\mu ,\pi }\;, \end{array}$$

(27) $$\begin{array}{ccc} A_{w} & = & -{\left(\frac{\partial Q_{z}}{\partial \mu }\right)}_{\theta ,\pi }\;, \end{array}$$

(28) $$\begin{array}{ccc} A_{p} & = & -{\left(\frac{\partial Q_{z}}{\partial \pi }\right)}_{\theta ,\mu }\;. \end{array}$$

We have finally arrived at defining the volumetric flow rate $Q_{p}$ in Equation (11),

(29) $$Q_{p}\left(\theta ,A_{w},A_{p}\right)=Q_{z}\left(\theta ,\mu ,\pi \right)+\mu A_{w}+\pi A_{p}\;,$$

and the relation between $Q_{u}$ and $Q_{p}$ is then

(30) $$Q_{p}\left(\theta ,A_{w},A_{p}\right)=Q_{u}\left(S,A_{w},A_{p}\right)-S\theta \;.$$

We see that θ (Equation (22)) plays the same role as temperature in ordinary thermodynamics. Hansen et al. [9] named it *agiture*, which stands for *agitation temperature*. They also named μ (Equation (27)) the *flow derivative*. It is analogous to *chemical potential* in ordinary thermodynamics. Lastly, π, defined in Equation (28), they called the *flow pressure*. It has no equivalent in ordinary thermodynamics. We note that the unit of the agiture is flow rate, whereas the unit of the flow derivative and the flow pressure is velocity.

## 5. Extensive and Intensive Variables

The variables $Q_{u}$, $Q_{z}$, $Q_{p}$, $A_{w}$, $A_{p}$, and *S* are extensive in the area of the REA: double its area *A*, and all these variable double. On the other hand, the variables θ, μ, and π are intensive. They do not change when *A* is changed.

We rescale $Q_{p}$, $A_{w}$, and $A_{p}$ by *A*, defining

(31) $$\begin{array}{ccc} \phi & = & \frac{A_{p}}{A}\;, \end{array}$$

(32) $$\begin{array}{ccc} S_{w}\phi & = & \frac{A_{w}}{A}=\frac{A_{w}}{A_{p}}\phi \;, \end{array}$$

(33) $$\begin{array}{ccc} v_{d} & = & v_{p}\phi =\frac{Q_{p}}{A}=\frac{Q_{p}}{A_{p}}\phi \;, \end{array}$$

(34) $$\begin{array}{ccc} s & = & \frac{S}{A}\;, \end{array}$$

where ϕ is the porosity, $S_{w}$ the wetting saturation, $v_{d}$ the Darcy velocity, $v_{p}$ the pore or seeping velocity, and *s* the entropy density.

In order to keep track of what happens to the variables in, e.g., $Q_{p}\left(\theta ,A_{w},A_{p},A\right)$, we express the extensivity through the scaling relation

(35) $$\lambda Q_{p}\left(\theta ,A_{w},A_{p},A\right)=Q_{p}\left(\theta ,\lambda A_{w},\lambda A_{p},\lambda A\right)\;.$$

We set the scale factor λ=1/A, finding

(36) $$v_{d}\left(\theta ,S_{w},\phi \right)=v_{p}\left(\theta ,S_{w},\phi \right)\phi =\frac{1}{A}Q_{p}\left(\theta ,A_{w},A_{p},A\right)=Q_{p}\left(\theta ,S_{w}\phi ,\phi ,1\right)\;.$$

Using the same arguments, we find that

(37) $$s=s(\theta ,S_{w},\phi )\;.$$

## 6. Emergent Variables

The three variables θ, μ, and π emerge as a result of the scale-up process. They are the conjugate variables to the entropy *S*, the wetting area $A_{w}$, and the pore area $A_{p}$, respectively. How are we to interpret them?

**Figure 2:**
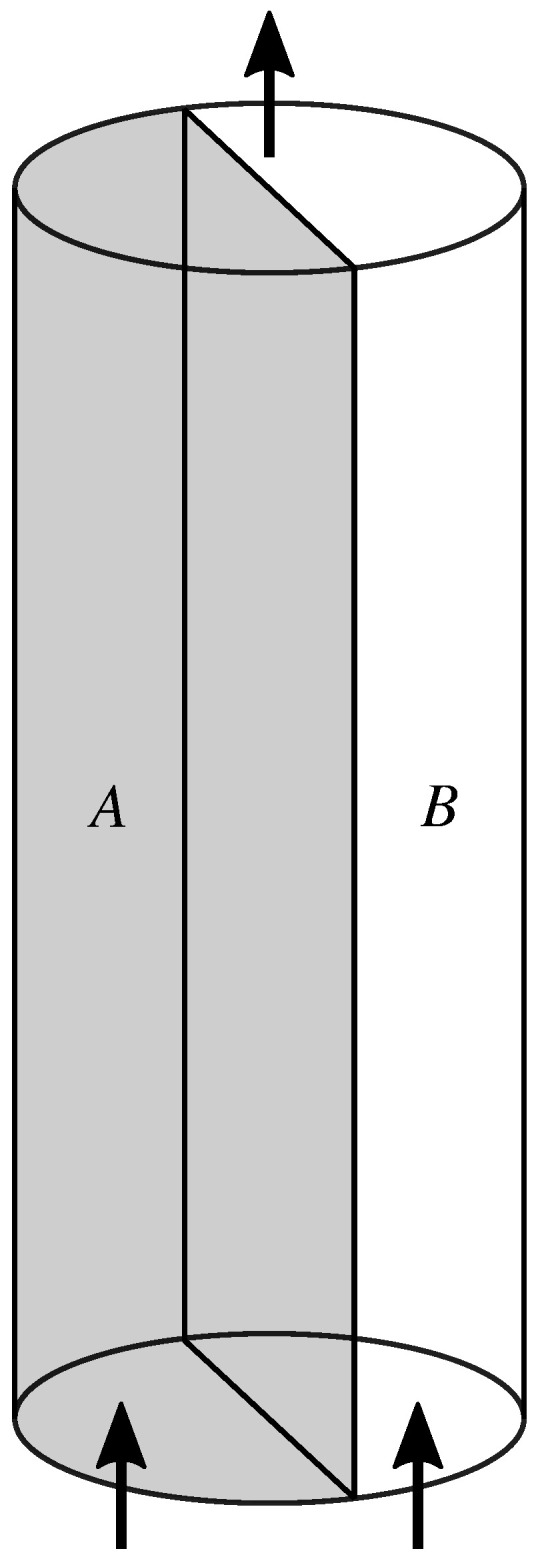
A cylindrical porous media sample consisting of two halves, A and B, having different properties with respect to the two immiscible fluids injected at the bottom edge. The two halves are in direct contact and fluids may pass unrestricted between the two halves.

We follow Hansen et al. [9], and consider a cylindrical porous medium (see Figure 2). We imagine a plane running through the cylinder axis parallel to the average flow direction. On one side of the plane, the porous medium has one set of properties; on the other side of the plane, it has another set of properties. The difference may be in chemical composition of the matrix, or it may consist of different porosity or topological structure. We name the two halves “A” and “B”, respectively. We assume the two halves to be statistically homogeneous along the axis.

We now assume that two immiscible and incompressible fluids are simultaneously pushed through the porous cylinder. Away from the edge where the two fluids are injected, the flow is in a steady state. This means that the flow statistics is invariant along the axis. However, it does not mean that the fluid–fluid interfaces do not move. At sufficient flow rates, they do and as a result, fluid clusters will merge and break up.

As the statistical distributions describing the flow is invariant along the flow axis, the configurational entropy *S* is invariant along this axis. As shown by Hansen et al. [9], this means that the agiture in sectors A and B, $\theta^{A}$ and $\theta^{B}$, are equal,

(38) $$\theta^{A}=\theta^{B}\;.$$

In ordinary thermodynamics, the rule is that the conjugate of a conserved quantity is constant in a heterogeneous system at equilibrium. This is a generalization of the argument for the temperature being the same everywhere in a system at equilibrium as the entropy is conserved and the temperature is its conjugate. This reasoning may be repeated for the flow problem.

Neither $A_{w}$ nor $A_{p}$ are conserved along the flow axis at the pore scale. This would imply that $\mu^{A}\neq \mu^{B}$ and $\pi^{A}\neq \pi^{B}$. This remains true at the continuum scale for $A_{p}$. However, $A_{w}$ is conserved at the continuum scale; see Equation (7). This is an emergent conservation law. We therefore conjecture that

(39) $$\mu^{A}=\mu^{B}\;.$$

### 6.1. Agiture

The agiture θ plays the role of a temperature in the thermodynamics-like formalism that we are developing. But, can we express it in terms of more familiar variables?

We note first that in the formalism we have developed in Section 4 and Section 5, there is one variable that is missing: the pressure *P*. Intuitively, one would expect the agiture to be related to the pressure gradient ∇P: a higher pressure gradient should indicate a higher agiture.

**Figure 3:**
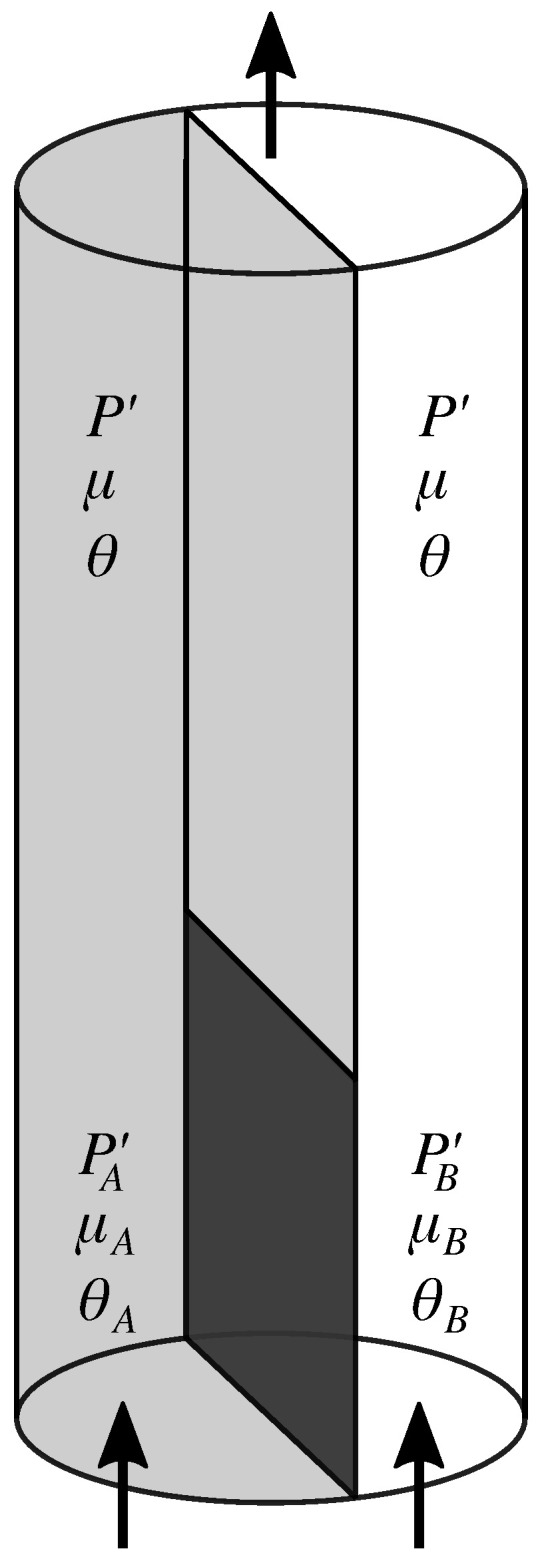
A cylindrical porous media sample consisting of two halves, A and B, having different properties with respect to the two immiscible fluids injected at the bottom edge. There is a impenetrable wall separating the two halves at the bottom third of the boundary. This is indicated by the dark section. Above, the two halves are in direct contact, as in Figure 2. A mixture of the two fluids are injected into the two halves at the lower edge. The pressure gradient is $P_{A}^{\prime }$ on the A side and $P_{B}^{\prime }$ on the B side up to the upper edge of the impenetrable wall. Likewise the flow derivatives are $\mu_{A}$ and $\mu_{B}$ and the agitures are $\theta_{A}$ and $\theta_{B}$. Higher up, where the two halves communicate, the pressure gradients, the flow derivatives, and the agitures become pairwise equal.

Figure 3 shows a cylindrical porous medium consisting of two halves A and B. They have different properties such as porosity, chemical composition of the matrix, or the pore spaces have different topology. At the bottom one third of the porous cylinder, the boundary between the two halves is impenetrable. This is indicated by the dark section of the boundary. Above, the two halves are in direct contact. We inject the immiscible fluids into each of the halves A and B at the bottom. We may perform this by injecting at different volumetric flow rates $Q_{p}^{A}=Q_{w}^{A}+Q_{n}^{A}$ and $Q_{p}^{B}=Q_{w}^{B}+Q_{n}^{B}$. This gives rise to flow derivatives $\mu_{A}$ and $\mu_{B}$, and agitures $\theta_{A}$ and $\theta_{B}$ in the two halves far enough from the inlet for the flow to be in a steady state. The pressure gradient will then point along the axis of the cylinder, the *z*-direction, and we will in the following use the notation $P^{\prime }=\partial P/\partial z$ for the gradient. We find a pressure gradient $P_{A}^{\prime }$ and $P_{B}^{\prime }$ in A and B, respectively. Further into the cylindrical sample above the impenetrable wall, the flow will adjust to a new steady state characterized by equal agiture θ and flow derivative μ, and in both halves according to Equations (38) and (39). Likewise the pressure gradient $P^{\prime }$ must be equal in the two halves since, there cannot be any net flow in either direction across the boundary between the two halves, apart from local fluctuations.

Since the variables, θ and μ on one side, and $P^{\prime }$ on the other side, form alternate descriptions, we must assume that they are related. The simplest relation between them we may write down is

(40) $$P^{\prime }=-c_{\theta }\theta +c_{\mu }\mu \;.$$

The reason for the minus sign in front of the θ term is due to the flow direction is the opposite of the pressure gradient direction. We note that the unit of $c_{\theta }$ is viscosity, and that of $c_{\mu }$ is viscosity times area.

We note that Equation (36) defines the seepage velocity $v_{p}\left(\theta ,S_{w},\phi \right)$ where $S_{w}$ rather than the flow derivative μ is a control variable. At the same time, the seepage velocity depends on the local pressure gradient, $v_{p}\left(P^{\prime },S_{w},\phi \right)$. Hence, we conjecture that $c_{\mu }=0$, so that

(41) $$P^{\prime }=-c_{\theta }\theta \;.$$

We note that $Q_{p}=Q_{p}\left(\theta ,A_{w},A_{p}\right)$, defined in Equation (29), may be written as

(42) $$Q_{p}=Q_{p}\left(P^{\prime },A_{w},A_{p}\right)=A_{p}v_{p}\left(P^{\prime },S_{w},\phi \right)\;,$$

where we have also used Equation (36). We see that this is a constitutive equation relating seepage velocity to local pressure gradient, saturation, and porosity.

To be concrete, we return for a moment to Section 2 and the constitutive equations forming the core of relative permeability theory, (1) and (2), which give

(43) $$v_{p}\left(P^{\prime },S_{w},\phi \right)=-\frac{K}{\phi }\left[\frac{k_{rw}\left(S_{w}\right)}{\mu_{w}}+\frac{k_{rn}\left(S_{w}\right)}{\mu_{n}}\right]P^{\prime }\;.$$

The agiture is the conjugate variable to the entropy. We may express it as

(44) $$\theta ={\left(\frac{\partial Q_{u}}{\partial S}\right)}_{A_{w},A_{p}}\;,$$

The entropy is given by

(45) $$S=-{\left(\frac{\partial Q_{p}}{\partial \theta }\right)}_{A_{w},A_{p}}=c_{\theta }{\left(\frac{\partial Q_{p}}{\partial P^{\prime }}\right)}_{A_{w},A_{p}}\;,$$

or, in terms of the entropy density,

(46) $$s=c_{\theta }{\left(\frac{\partial v_{p}}{\partial P^{\prime }}\right)}_{S_{w},\phi }\;.$$

We note that the *differential mobility* is defined as

(47) $$m\left(P^{\prime },S_{w},\phi \right)=-{\left(\frac{\partial v_{p}}{\partial P^{\prime }}\right)}_{S_{w},\phi }\;.$$

Using the relative permeability constitutive Equation (43), we find the mobility in this case to be

(48) $$m\left(S_{w},\phi \right)=\frac{K}{\phi }\left[\frac{k_{rw}\left(S_{w}\right)}{\mu_{w}}+\frac{k_{rn}\left(S_{w}\right)}{\mu_{n}}\right]\;.$$

Mobility and differential mobility are equal in this case, as the velocity is linear in the pressure gradient.

Comparing Equations (46) and (47), we see that

(49) $$s=-c_{\theta }m\;.$$

Since we are dealing with *differential entropy*, it is not a problem that it is negative. It is surprising that the differential mobility and the entropy should be related.

Equation (41) relates the agiture θ and the pressure gradient $P^{\prime }$ and the way we have argued is by demonstrating that the pressure gradient behaves as one would expect an agiture (=agitation temperature) should do. However, one could test Equation (41) directly by measuring the left hand side and compare it to the right hand side. This is difficult, as it would entail measuring the configurational entropy and then using Equation (44), and measuring entropy is also difficult in ordinary thermodynamics.

### 6.2. Flow Derivative

Hansen et al. [48] defined the two *thermodynamic velocities* as

(50) $$\begin{array}{c} {\hat{v}}_{w}={\left(\frac{\partial Q_{p}}{\partial A_{w}}\right)}_{\theta ,A_{n}}\;, \end{array}$$

(51) $$\begin{array}{c} {\hat{v}}_{n}={\left(\frac{\partial Q_{p}}{\partial A_{n}}\right)}_{\theta ,A_{w}}\;, \end{array}$$

where the control variables are θ, $A_{w}$, and $A_{n}$, defined in (12), making $A_{p}$ a dependent variable. Changing the variables $\left(\theta ,A_{w},A_{n}\right)\to \left(\theta ,S_{w},A_{p}\right)$, these two equations may be written as

(52) $$\begin{array}{c} {\hat{v}}_{w}=v_{p}+S_{n}{\left(\frac{\partial v_{p}}{\partial S_{w}}\right)}_{\theta ,\phi }\;, \end{array}$$

(53) $$\begin{array}{c} {\hat{v}}_{n}=v_{p}-S_{w}{\left(\frac{\partial v_{p}}{\partial S_{w}}\right)}_{\theta ,\phi }\;. \end{array}$$

The flow derivative μ, which is the conjugate of the wetting area $A_{w}$, and thereby also the saturation $S_{w}$, is given by

(54) $$\mu =-{\left(\frac{\partial Q_{p}}{\partial A_{w}}\right)}_{\theta ,A_{p}}={\left(\frac{\partial v_{p}}{\partial S_{w}}\right)}_{\theta ,\phi }\;.$$

Hence, Equation (53) may be recognized as a Legendre transformation substituting $S_{w}\to \mu$,

(55) $${\hat{v}}_{n}\left(\theta ,\mu ,\phi \right)=v_{p}\left(\theta ,S_{w},\phi \right)-S_{w}\left(\theta ,\mu ,\phi \right)\mu \;.$$

In other words, the non-wetting thermodynamic velocity is the Legendre transformation of the average seepage velocity with respect to the saturation.

### 6.3. Flow Pressure

The flow pressure π is the conjugate of the pore area $A_{p}$, and thereby the porosity ϕ,

(56) $$\pi =-{\left(\frac{\partial Q_{p}}{\partial A_{p}}\right)}_{\theta ,A_{w}}={\left(\frac{\partial v_{p}}{\partial \phi }\right)}_{\theta ,S_{w}}\;.$$

There are no conservation laws associated with this variable.

We expect the flow pressure π to be of less practical use, as we expect that the flow rate $Q_{p}$ to be proportional to the pore area $A_{p}$. This makes the Legendre transform between $A_{p}$ and π break down due to lack of convexity. The flow pressure will nevertheless play a role, e.g., when porosity gradients are present.

## 7. Seepage Velocities and the Co-Moving Velocity

The thermodynamic velocities defined in Equations (50) and (51) are not the seepage velocities, which we define as

(57) $$\begin{array}{c} v_{w}=\frac{Q_{w}}{A_{w}}\;, \end{array}$$

(58) $$\begin{array}{c} v_{n}=\frac{Q_{n}}{A_{n}}\;. \end{array}$$

These are the velocities that measured in the laboratory, whereas the thermodynamic velocities are not. We rewrite Equation (11) as

(59) $$Q_{p}=Q_{w}+Q_{n}=v_{w}A_{w}+v_{n}A_{n}\;.$$

We express the saturations in terms of the variables $A_{w}$ and $A_{n}$,

(60) $$S_{w}=\frac{A_{w}}{A_{w}+A_{n}}\;,$$

and

(61) $$S_{n}=\frac{A_{n}}{A_{w}+A_{n}}\;,$$

leading to Equation (3). We may then write Equation (59) as

(62) $$v_{p}=v_{w}S_{w}+v_{n}S_{n}\;,$$

where $v_{p}$, the average velocity, is defined in Equation (36).

We now turn to the thermodynamic velocities, Equations (50) and (51). The average flow rate $Q_{p}$ obeys the Euler scaling relation,

(63) $$\lambda Q_{p}\left(P^{\prime },A_{w},A_{n}\right)=Q_{p}\left(P^{\prime },\lambda A_{w},\lambda A_{n}\right)\;.$$

We then have from the Euler theorem for homogeneous functions,

(64) $$Q_{p}={\left(\frac{\partial Q_{p}}{\partial A_{w}}\right)}_{A_{n},\phi ,P^{\prime }}A_{w}+{\left(\frac{\partial Q_{p}}{\partial A_{n}}\right)}_{A_{w},\phi ,P^{\prime }}A_{n}={\hat{v}}_{w}A_{w}+{\hat{v}}_{n}A_{n}\;.$$

Dividing this equation by $A_{p}$ gives

(65) $$v_{p}={\hat{v}}_{w}S_{w}+{\hat{v}}_{n}S_{n}\;.$$

Comparing Equations (62) and (65),

(66) $$v_{p}=v_{w}S_{w}+v_{n}S_{n}={\hat{v}}_{w}S_{w}+{\hat{v}}_{n}S_{n}\;.$$

This equation does not imply that $v_{w}={\hat{v}}_{w}$ and $v_{n}={\hat{v}}_{n}$. Rather, the most general relation between the thermodynamic and seeping velocities are

(67) $$\begin{array}{c} v_{w}={\hat{v}}_{w}-S_{n}v_{m}\;, \end{array}$$

(68) $$\begin{array}{c} v_{n}={\hat{v}}_{n}+S_{w}v_{m}\;, \end{array}$$

where $v_{m}$ is the *co-moving velocity* [48,49,50]. We may combine these two equations with Equations (52) and (53) to find

(69) $$\begin{array}{ccc} v_{w} & = & v_{p}+S_{n}\left[{\left(\frac{\partial v_{p}}{\partial S_{w}}\right)}_{\phi ,P^{\prime }}-v_{m}\right]\;, \end{array}$$

(70) $$\begin{array}{ccc} v_{n} & = & v_{p}-S_{w}\left[{\left(\frac{\partial v_{p}}{\partial S_{w}}\right)}_{\phi ,P^{\prime }}-v_{m}\right]\;. \end{array}$$

By taking the derivative of Equation (66) with respect to $S_{w}$, and using Equations (67) and (68), we find

(71) $$v_{m}={\left(\frac{\partial v_{p}}{\partial S_{w}}\right)}_{\phi ,P^{\prime }}-v_{w}+v_{n}=S_{w}{\left(\frac{\partial v_{w}}{\partial S_{w}}\right)}_{\phi ,P^{\prime }}+S_{n}{\left(\frac{\partial v_{n}}{\partial S_{w}}\right)}_{\phi ,P^{\prime }}\;.$$

We now return to Equation (55), which may now be expressed in terms of the seepage velocity $v_{n}$, rather than the thermodynamic velocity ${\hat{v}}_{n}$,

(72) $$v_{n}\left(\theta ,\mu ,\phi \right)-S_{w}\left(\theta ,\mu ,\phi \right)v_{m}\left(\theta ,\mu ,\phi \right)=v_{p}\left(\theta ,S_{w},\phi \right)-S_{w}\left(\theta ,\mu ,\phi \right)\mu \;,$$

signifying that (θ,μ,ϕ) are the natural variables for the co-moving velocity. Measurements of the co-moving velocity suggest that it has the simple functional form [51,52,53],

(73) $$v_{m}\left(\theta ,\mu ,\phi \right)=a\left(\theta ,\phi \right)+b\left(\theta ,\phi \right)\mu \;,$$

based on analysis of relative permeability data, on dynamic pore network modeling, and on lattice Boltzmann simulations on reconstructed sandstones.

It has recently been realized that the co-moving velocity has an equivalent in the thermodynamics of two-fluid mixtures [54], where a *co-molar volume* ties the partial molar volumes of a binary mixture together with the Voronoi volumes of each of the fluids in the mixture. When plotting the co-molar volume against the variable corresponding to μ, it is *nearly* linear, but not quite.

We note that the thermodynamic velocities $\left({\hat{v}}_{w},{\hat{v}}_{n}\right)$ can be found by knowing $v_{p}$ alone; see Equations (52) and (53). The seepage velocities, on the other hand, requires additional knowledge, i.e., $v_{p}$ and $v_{m}$, which are given by Equations (69) and (70). They provide a two-way mapping,

(74) $$\left(\begin{array}{c} v_{w}\\ v_{n} \end{array}\right)↔\left(\begin{array}{c} v_{p}\\ v_{m} \end{array}\right)\;.$$

## 8. Control Variables

In Section 4, we made a choice when focusing on the averages $Q_{u}$, $A_{w}$, and $A_{p}$ in Equations (14) to (16), leading to a thermodynamics-like formalism based upon the extensive variables $Q_{p}$, $A_{w}$, and $A_{p}$, and their intensive conjugates θ ($=-c_{\theta }P^{\prime }$), μ, and π. In core flooding, these are not the natural control variables. Rather, one controls the flow rates $Q_{w}$ and $Q_{n}$ into the core, making $A_{w}$ (i.e., the saturation) and the pressure gradient the dependent variables. It is possible to build a Jaynes statistical mechanics based on $Q_{u}$, $Q_{w}$, and $A_{p}$, rather than $Q_{u}$, $A_{w}$, and $A_{p}$. We will do this elsewhere. However, we can still develop the ensuing thermodynamics-like formalism as long as we work only with the extensive variables. We will do so in the following.

### Controlling the Fractional Flow Rate

Our starting point is again the REA shown in Figure 1, and Equations (11) and (12). We now assume that our control variables are $Q_{w}$ and $Q_{n}$ together with $A_{p}$, the REA pore space. The dependent variable is the pressure gradient $P^{\prime }$. They are related through the constitutive equation

(75) $$P^{\prime }=P^{\prime }\left(Q_{w},Q_{n},A_{p}\right)\;.$$

We may rescale the extensive variables in this expression, $A_{p}\to \lambda A_{p}$, $Q_{w}\to \lambda Q_{w}$, and $Q_{n}\to \lambda Q_{n}$. In practice, changing $A_{p}$ means changing the sample. Hence, this is a theoretical exercise. Nevertheless, mathematics works. This leaves $P^{\prime }$ unchanged, i.e.,

(76) $$P^{\prime }\left(\lambda Q_{w},\lambda Q_{n},\lambda A_{p}\right)=P^{\prime }\left(Q_{w},Q_{n},A_{p}\right)\;.$$

It is, however, convenient to rewrite Equation (75) as

(77) $$A_{p}=A_{p}\left(P^{\prime },Q_{w},Q_{n}\right)\;,$$

making it implicit with respect to $P^{\prime }$. The scaling relation (76) then takes the form

(78) $$\lambda A_{p}\left(P^{\prime },Q_{w},Q_{n}\right)=A_{p}\left(P^{\prime },\lambda Q_{w},\lambda Q_{n}\right)\;.$$

We then use the Euler theorem for homogeneous functions,

(79) $$A_{p}={\left(\frac{\partial A_{p}}{\partial Q_{w}}\right)}_{Q_{n},\phi ,P^{\prime }}Q_{w}+{\left(\frac{\partial A_{p}}{\partial Q_{n}}\right)}_{Q_{w},\phi ,P^{\prime }}Q_{n}={\hat{p}}_{w}Q_{w}+{\hat{p}}_{n}Q_{n}\;,$$

where we have defined the *thermodynamic paces*,

(80) $${\hat{p}}_{w}={\left(\frac{\partial A_{p}}{\partial Q_{w}}\right)}_{Q_{n},\phi ,P^{\prime }}\;,$$

and

(81) $${\hat{p}}_{n}={\left(\frac{\partial A_{p}}{\partial Q_{n}}\right)}_{Q_{w},\phi ,P^{\prime }}\;.$$

Pace is inverse velocity. We also define the *physical paces*,

(82) $$p_{p}=\frac{A_{p}}{Q_{p}}=\frac{1}{v_{p}}\;,$$

(83) $$p_{w}=\frac{A_{w}}{Q_{w}}=\frac{1}{v_{w}}\;,$$

and

(84) $$p_{n}=\frac{A_{n}}{Q_{n}}=\frac{1}{v_{n}}\;.$$

This allows us to write Equation (12) as

(85) $$A_{p}=p_{w}Q_{w}+p_{n}Q_{n}\;.$$

Combining this equation with Equation (79) gives

(86) $$A_{p}=p_{w}Q_{w}+p_{n}Q_{n}={\hat{p}}_{w}Q_{w}+{\hat{p}}_{n}Q_{n}\;.$$

We now introduce the fractional flow rates,

(87) $$F_{w}=\frac{Q_{w}}{Q_{w}+Q_{n}}\;,$$

and

(88) $$F_{n}=\frac{Q_{n}}{Q_{w}+Q_{n}}\;,$$

so that

(89) $$F_{w}+F_{n}=1\;.$$

We may then write Equation (86) as

(90) $$p_{p}=p_{w}F_{w}+p_{n}F_{n}={\hat{p}}_{w}F_{w}+{\hat{p}}_{n}F_{n}\;,$$

where $p_{p}$, the average pace, is defined in Equation (82).

We now make a coordinate transformation,

(91) $$\left(P^{\prime },Q_{w},Q_{n}\right)\to \left(P^{\prime },Q_{p},F_{w}\right)\;,$$

so that we have

(92) $$A_{p}\left(P^{\prime },Q_{p},F_{w}\right)=A_{p}\left(P^{\prime },Q_{w},Q_{n}\right)\;.$$

We use extensivity,

(93) $$\lambda A_{p}\left(P^{\prime },Q_{p},F_{w}\right)=A_{p}\left(P^{\prime },\lambda Q_{p},F_{w}\right)\;,$$

leading to

(94) $$A_{p}\left(P^{\prime },Q_{p},F_{w}\right)=Q_{p}A_{p}\left(P^{\prime },1,F_{w}\right)=Q_{p}p_{p}\left(P^{\prime },F_{w}\right)\;,$$

when setting $\lambda =1/Q_{p}$ and defining $p_{p}\left(P^{\prime },F_{w}\right)=A_{p}\left(P^{\prime },1,F_{w}\right)$.

We express the thermodynamic paces Equations (80) and (81) in terms of $\left(P^{\prime },Q_{p},F_{w}\right)$, finding

(95) $${\hat{p}}_{w}=p_{p}+F_{n}{\left(\frac{\partial p_{p}}{\partial F_{w}}\right)}_{\phi ,P^{\prime }}\;,$$

and

(96) $${\hat{p}}_{n}=p_{p}-F_{w}{\left(\frac{\partial p_{p}}{\partial F_{w}}\right)}_{\phi ,P^{\prime }}\;.$$

We may define a *co-moving pace function*, $p_{m}$, by the expressions

(97) $${\hat{p}}_{w}=p_{w}+F_{n}p_{m}\;,$$

and

(98) $${\hat{p}}_{n}=p_{n}-F_{w}p_{m}\;,$$

where $p_{m}$ is the most general function to fulfill Equation (90). Combining these two equations with Equations (96) and (97) gives

(99) $$\begin{array}{ccc} p_{w} & = & p_{p}+F_{n}\left[{\left(\frac{\partial p_{p}}{\partial F_{w}}\right)}_{\phi ,P^{\prime }}-p_{m}\right]\;, \end{array}$$

(100) $$\begin{array}{ccc} p_{n} & = & p_{p}-F_{w}\left[{\left(\frac{\partial p_{p}}{\partial F_{w}}\right)}_{\phi ,P^{\prime }}-p_{m}\right]\;, \end{array}$$

By taking the derivative of Equation (90) with respect to $F_{w}$, and using Equations (97) and (98), we find

(101) $$p_{m}={\left(\frac{\partial p_{p}}{\partial F_{w}}\right)}_{\phi ,P^{\prime }}-p_{w}+p_{n}=F_{w}{\left(\frac{\partial p_{w}}{\partial F_{w}}\right)}_{\phi ,P^{\prime }}+F_{n}{\left(\frac{\partial p_{n}}{\partial F_{w}}\right)}_{\phi ,P^{\prime }}\;.$$

**Figure 4:**
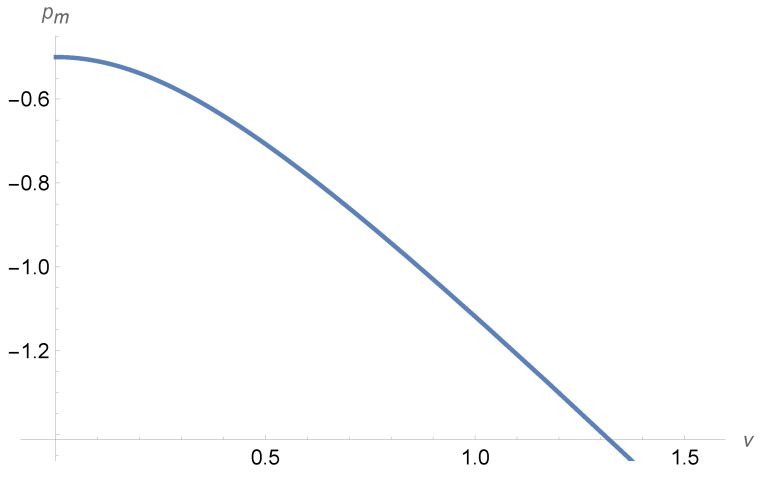
The co-moving pace $p_{m}$ against ν for the Picchi and Battiato pair of relative permeabilities [15]; Equations (106) and (107) with $\mu_{w}=\mu_{n}$, $k_{rw}^{0}=k_{rn}^{0}/2$, and α=1/2.

We may at this point ask whether the co-moving pace $p_{m}$ is a linear function in the variable

(102) $$\nu ={\left(\frac{\partial p_{p}}{\partial F_{w}}\right)}_{\phi ,P^{\prime }}$$

in the same way that the co-moving velocity $v_{m}$ is a linear function of $\mu ={\left(\partial v_{p}/\partial S_{w}\right)}_{\phi ,P^{\prime }}$; see Equation (73). The answer is no. This may, for example, be seen by testing the relative permeability pair proposed by Picchi and Battiato [15],

(103) $$\begin{array}{ccc} k_{rw} & = & k_{rw}^{0}S_{w}^{2}\;, \end{array}$$

(104) $$\begin{array}{ccc} k_{rn} & = & k_{rn}^{0}S_{n}^{2}\left[1+2\frac{\alpha \mu_{n}}{\mu_{w}}\frac{S_{w}}{S_{n}}\right]\;, \end{array}$$

where 0≤α≤1 is a parameter. This pair leads to the co-moving velocity

(105) $$v_{m}=\alpha k_{rn}^{0}+\frac{\mu }{2}\;,$$

in units of $v_{0}=-KP^{\prime }/\mu_{w}\phi$. The corresponding co-moving pace is a complex curve when plotting $p_{m}$ vs. ν; see Figure 4.

We note that we used the following relations between fractional flow $F_{w}$ and saturation $S_{w}$ in calculating $p_{m}$ above,

(106) $$F_{w}=\frac{Q_{w}}{Q_{p}}=\frac{A_{p}S_{w}v_{w}}{A_{p}v_{p}}=S_{w}\;\frac{v_{w}\left(P^{\prime },S_{w},\phi \right)}{v_{p}\left(P^{\prime },S_{w},\phi \right)}\;.$$

Likewise, we have

(107) $$S_{w}=\frac{A_{w}}{A_{p}}=\frac{Q_{p}F_{w}p_{w}}{Q_{p}p_{p}}=F_{w}\;\frac{p_{w}\left(P^{\prime },F_{w},\phi \right)}{p_{p}\left(P^{\prime },F_{w},\phi \right)}\;.$$

## 9. Discussion and Conclusions

The aim of this review has been to present the central ideas of the thermodynamics-like formalism for immiscible two-phase flow in porous media that was first introduced by Hansen et al. [48] in the context of steady-state flow. However, the ultimate goal of this approach is to be able to handle flow that is not in a steady state. This means handling gradients in the conjugate variables to configurational entropy, saturation and porosity: agiture θ, flow derivative μ, and flow pressure π, turning the equilibrium thermodynamics-like formalism into a non-equilibrium thermodynamics-like formalism.

We noted in Section 4 that actually calculating the partition function (Equation (19)) may be impossible in the present context. The consequence of this is that we are unable to derive directly the constitutive equation $Q_{p}\left(P^{\prime },A_{w},A_{p}\right)$ from the pore-level physics. It must be found by other means, and then used as input in the thermodynamics-like formalism.

We have conjectured here that the agiture, the temperature-like emergent variable conjugate to the configurational entropy, is proportional to the pressure gradient. A surprising consequence of this conjecture is that the differential mobility of the fluids is directly related to the entropy: higher differential mobility means lower configurational entropy.

The question of what are the control variables and the dependent variables is central, and much of the thermodynamics-like formalism concerns how to switch between the extensive variables $Q_{p}$, $A_{w}$, and $A_{p}$, and their intensive conjugates, θ, μ, and π. However, there was already a choice made when the three extensive variables were picked. We could have made other choices. In this review, we discuss, for the first time, another choice: $Q_{p}$, $Q_{w}$ and $A_{p}$. Without developing a Jaynes-type statistical mechanics based on this choice, we nevertheless explore aspects of the ensuing thermodynamics-like formalism. We find a set of equations that parallel those found in [48], but where the fractional flow rate $F_{w}$ rather than the saturation $S_{w}$ is a control variable. Since this is the natural variable in core flooding experiments, not the saturation, such a formalism is necessary.

## Data Availability

The original contributions presented in this study are included in the article. Further inquiries can be directed to the corresponding author.
